# Magnetic resonance bone imaging: applications to vertebral lesions

**DOI:** 10.1007/s11604-023-01449-4

**Published:** 2023-05-20

**Authors:** Kazuhiro Tsuchiya, Miho Gomyo, Shichiro Katase, Sayuki Hiraoka, Hidekatsu Tateishi

**Affiliations:** 1 Department of Radiology, JR Tokyo General Hospital, 2-1-3 Yoyogi, Shibuya-ku, Tokyo, 151-8528 Japan; 2 Department of Radiology, Faculty of Medicine, Kyorin University, 6-20-2 Shinkawa, Mitaka City, Tokyo 181-8611 Japan

**Keywords:** Spine, MR imaging, Bone imaging, CT-like MRI

## Abstract

MR bone imaging is a recently introduced technique, that allows visualization of bony structures in good contrast against adjacent structures, like CT. Although CT has long been considered the modality of choice for bone imaging, MR bone imaging allows visualization of the bone without radiation exposure while simultaneously allowing conventional MR images to be obtained. Accordingly, MR bone imaging is expected as a new imaging technique for the diagnosis of miscellaneous spinal diseases. This review presents several sequences used in MR bone imaging including black bone imaging, ultrashort/zero echo time (UTE/ZTE) sequences, and T1-weighted 3D gradient-echo sequence. We also illustrate clinical cases in which spinal lesions could be effectively demonstrated on MR bone imaging, performed in most cases using a 3D gradient-echo sequence at our institution. The lesions presented herein include degenerative diseases, tumors and similar diseases, fractures, infectious diseases, and hemangioma. Finally, we discuss the differences between MR bone imaging and previously reported techniques, and the limitations and future perspectives of MR bone imaging.

## Introduction

As well known among radiologists, computed tomography (CT) depicts the internal structures of the bones and the presence of calcifications more clearly as compared with conventional MR imaging. Furthermore, the short examination time, the relatively low cost, and the easy access have long made CT the tool of choice for bone imaging. MR depiction of solid bone structures is challenging due to the low proton density (20% of water) and very short T2 relaxation time (about 390 μs at 3 T) [1, 2]. MR bone imaging (CT-like MRI), which provides contrast using a short echo time (TE) on the bone, is a new technology that has been developed in recent years and is not yet widely recognized. However, in contrast to CT, which is inevitably accompanied by exposure of the examinee to ionizing radiation, MR bone imaging is expected to be useful for the examination of various regions such as the bone, and adjacent soft tissue structures such as ligaments.

This review focuses on summarizing the current MR bone imaging techniques and their pros and cons by referring to the previous research. For that purpose, we first discuss the basic issues of MR bone imaging and then present our clinical experience with the technique for the diagnosis of vertebral lesions by describing our current imaging methods and illustrating representative cases.

## Basic issues of MR bone imaging

### ***Current imaging techniques in MR bone imaging (***Table 1***)***

**Table 1:** Features of three main techniques for MR bone imaging

Black bone imaging	Ultrashort/zero echo time sequences	3D gradient-echo sequences
Product names (if any)	UTE, ZTE, PETRA, FFE3D with UTE Single-Echo and Multi-Echo	FRACTURE, 3D FLASH, VIBE, 3D SPGR, LAVA, 3D T1-FFE, THRIVE, FFE 3D Single-Echo and Multi-Echo, FE 3D Single-Echo and Multi-Echo
Technique		
Indirect imaging of cortical bone Short TR/TE makes bone cortex hypointensity	Direct imaging of cortical bone High-speed switching between RF transmission and signal reception	Indirect imaging of cortical bone Short TR/TE with optimized flip angle
Pros		
More CT-like images compared with 3D gradient-echo sequences Commonly available Low hardware/software requirements	Most CT-like images compared with other techniques	Commonly available Low hardware/software requirements Good depiction of soft tissues (ligaments, muscles, tendons, etc.)
Cons		
Severe susceptibility artifacts	Not generally available High hardware/software requirements Moderate susceptibility artifacts Prone to motion artifacts	Severe susceptibility artifacts Prone to motion artifacts
Available manufacture		
Any vendor	GE Siemens Canon	Philips Siemens GE Canon

*UTE* ultrashort echo time, *ZTE* zero echo time, *PETRA* pointwise encoding time reduction with radial acquisition, *FRACTURE* fast field echo resembling a CT using restricted echo-spacing, *FLASH* fast low angle shot, *VIBE* volume incorporated breath-hold examination, SPGR spoiled gradient recalled acquisition in the steady state, *LAVA* liver acquisition with volume acceleration, *FFE* fast field echo, *THRIVE* T1-high resolution isotropic volume excitation, *TR* repetition time, *TE* echo time, *RF* radiofrequency

#### Black bone imaging

Black bone imaging is a gradient-echo (GRE) sequence that was first reported as an MR bone imaging technique that employs a short flip angle of 5° optimized for good contrast between bone and soft tissues and a short repetition time (TR)/TE [3]. The TE in black bone imaging is set to be longer than the T2 relaxation time of the bone, which makes black bone imaging a useful method for indirectly visualizing the bone by making only the bone cortex hypointense and the other tissues hyperintense in a uniform manner. After the gray-scale reversal of the images thus obtained, the cortical bones are visualized as hyperintensities, resulting in contrast like that in CT. However, in black bone grayscale-reversed images, areas such as air, where protons do not exist, and areas such as calcification and hemorrhage, which are visualized as hypointensities in images obtained using the gradient-echo sequence, are visualized as hyperintensities, just like cortical bone. Accordingly, the boundary between bone and air around the paranasal sinuses becomes unclear. The most significant advantage of this method is its high availability. Black bone imaging can be used with any scanner without the need to use advanced technologies such as ultrashort TE (UTE), which will be mentioned later, and without any dependence on magnetic field strength.

### Ultrashort/zero echo time sequences

Since the TE of black bone imaging is longer than the T2 relaxation time of the bone, it is a method for indirectly visualizing the bone. Imaging methods such as UTE with a TE of 8–50 μs and zero TE (ZTE) with a TE of almost zero have been developed as methods for direct imaging of the bones by the signal acquisition of the cortical bones [4, 5]. These methods have been made possible by advances in hardware, such as radiofrequency coils, which enable high-speed switching between transmission and reception, and the introduction of higher gradient magnetic fields. Although these methods collect signals directly from the cortical bones, other tissues with long T2 are also visualized as hyperintensities, like bones. Therefore, MR bone images are obtained by methods such as subtraction using multi-echo imaging [6], bias field correction, and inverse-logarithmic rescaling [7].

### 3D gradient-echo sequences

Recently, multi-TE MR bone imaging has been developed and become clinically available. Employing short TRs and TEs with optimized flip angles in a versatile T1-weighted 3D-GRE sequence, signals acquired multiple times at the same echo interval (in phase) are summed and gray-scale reversed with resultant contrast between the cortical and cancellous bones. GRE pulse sequences investigated for MR bone imaging include 3D FLASH (fast low angle shot) and VIBE (volumetric interpolated breath-hold examination) (Siemens Healthineers), 3D SPGR (spoiled gradient recalled acquisition in the steady state) and LAVA (liver acquisition with volume acceleration) (GE HealthCare), as well as 3D T1-FFE (fast field echo) and THRIVE (T1-high resolution isotropic volume excitation) (Philips Healthcare) [8–12].

Because these sequences do not generate contrast specific for cortical bones, some modified techniques have been proposed, including Dixon subtraction 3D FLASH and FRACTURE (fast field echo resembling a CT using restricted echo-spacing) (Philips Healthcare). FRACTURE, currently available on commercial scanners, is a Cartesian 3D GRE sequence that yields high spatial-resolution MR bone images. FRACTURE collects magnitude images from multiple in-phase echoes and summated them followed by subtraction with the final echo image [13–15].

### Other techniques

Other techniques so far investigated for MR bone imaging include the use of susceptibility-weighted imaging (SWI) and the application of deep learning [8, 16–18]. SWI-based MR bone imaging reportedly shows high diagnostic performance (sensitivity: 98.9%, specificity: 99.1%) in differentiating osteophyte and disc herniations [19]. Another method is synthetic CT images generated from MR data, like synthetic MR imaging [20]. This method has already been used in simulation and dose calculation for radiotherapy in patients with pelvic tumors such as prostate cancer.

### Imaging method used in this presentation

**Fig. 1:**
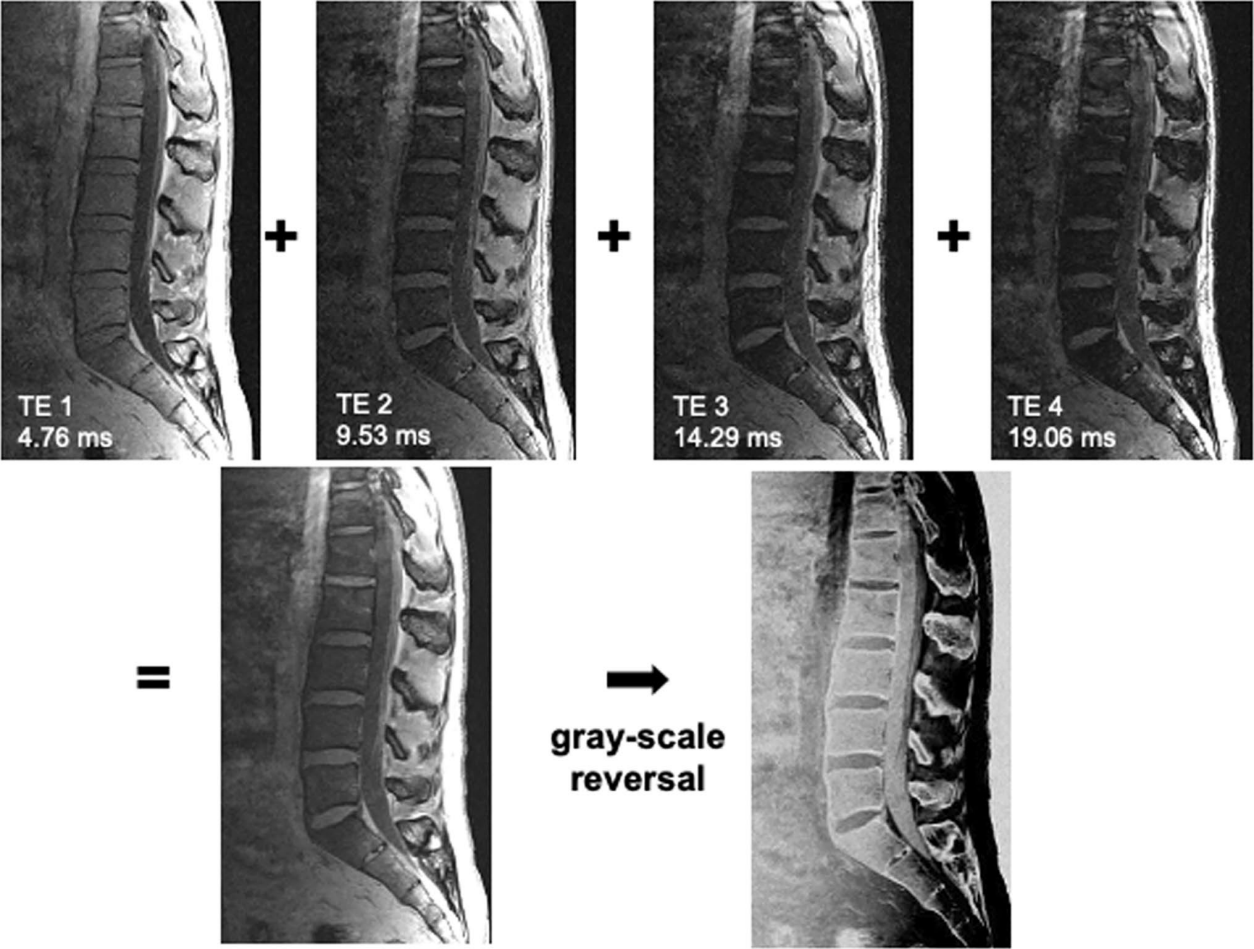
Postprocessing used in the 3D-GRE sequence. Signals from TEs of 4.76, 9.53, 14.29, and 19.06 ms are added followed by a gray-scale reversal to obtain MR bone images

Previous reports on MR bone imaging of the spine tended to involve UTE/ZTE sequences [21–24]. That is presumably because UTE/ZTE sequences allow direct visualization of bones, especially the cortical bones, resulting in preferable diagnostic feasibility. Meanwhile, the superiority of the T1-weighted 3D-GRE sequence for the diagnosis of vertebral fracture and degenerative changes has also been reported [25]. In the present article, our experience of applying the 3D-GRE sequence (volume incorporated breath-hold examination, VIBE) for the diagnosis of vertebral lesions is presented, unless otherwise specified (Table 2). For postprocessing, we added signals from TEs of 4.76, 9.53, 14.29, and 19.06 ms obtained at 1.5 T and performed gray-scale reversal to generate the MR bone images (Fig. 1). With this imaging method, data misregistration was not a problem except for patients with considerable body movement.

**Table 2:** Scanning parameters of the sequence used in this article

Scanner	Field strength	Sequence	
Avanto Fit (Siemens Healthineers)	1.5 T	3D-GRE sequence (VIBE)	
TR	TE	NEX	Flip angle
22 ms	4.76, 9.53, 14.29, 19. 06 ms	1	15
Field of view	In-plane spatial resolution	Slice thickness	Slab
300 mm	0.78 × 0.78 mm	0.7 mm	1
Imaging direction	PAT mode	Fat suppression	Scanning time
sagittal	GRAPPA	none	5 min 7 s

*GRE* gradient-echo, *VIBE* volumetric interpolated breath-hold examination, *PAT* parallel acquitition technique, *GRAPPA* generalized autocallibrating partially parallel acquisition

## Clinical applications

**Fig. 2:**
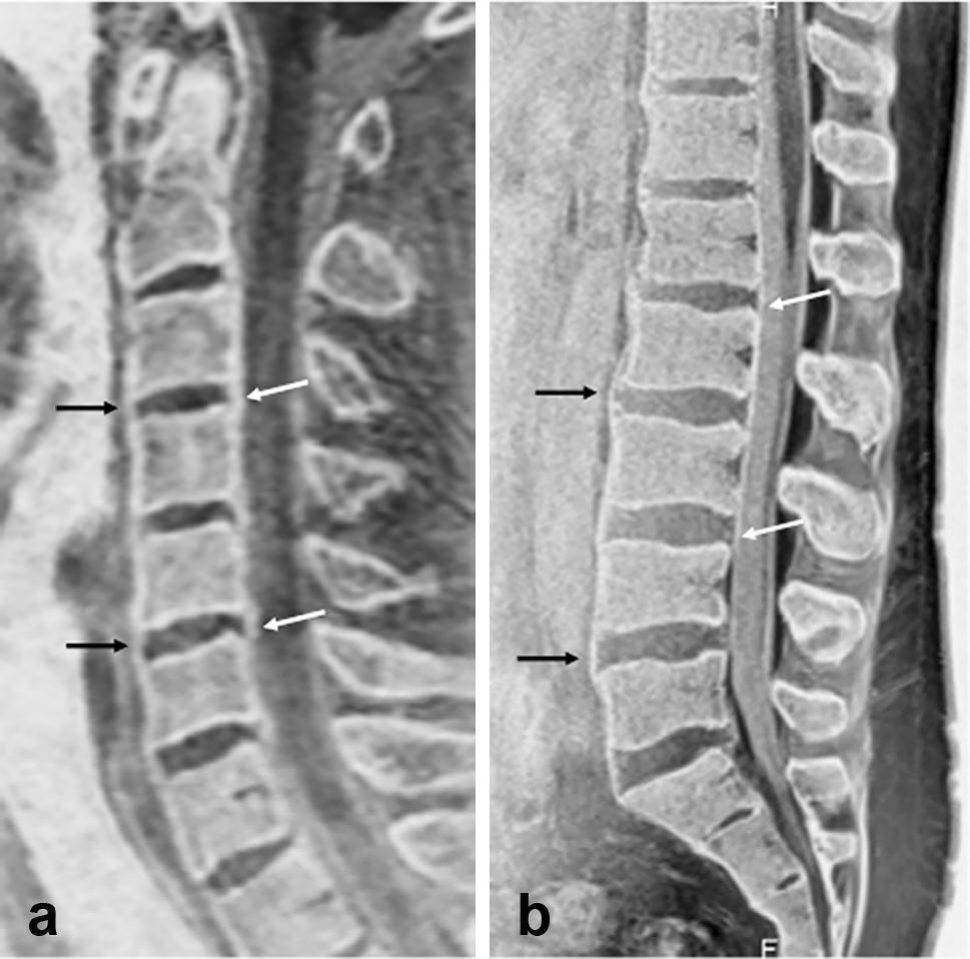
Normal cervical and lumbar spine. Midsagittal MR bone image (**a**) of a 36-year-old male shows a normal appearance of the cervical spine. The anterior and posterior longitudinal ligaments are depicted as smooth linear lines (black arrows and white arrows, respectively). Midsagittal MR bone image (**b**) of the lumbar spine of a 39-year-old male similarly shows the anterior and posterior longitudinal ligaments (black arrows and white arrows, respectively). The bone cortex and trabeculae are delineated well at the cervical and lumbar spines. At the posterior part of many vertebral bodies, exits of the basivertebral veins are visualized as triangular or linear hypointense structures. The spinal cord shows hypointensity relative to the cerebrospinal fluid within the dural sac

In this section, changes and disorders effectively depicted by MR bone imaging when added to conventional techniques are presented (Table 3). In the normal spine, MR bone images well delineate the endplates and the trabeculae of the vertebra. Additionally, the anterior and posterior longitudinal ligaments not usually identified on CT are depicted as smooth linear structures. The spinal cord, nerve roots, and cerebrospinal fluid are discriminated in the dural sac. Intervertebral discs are visualized showing mild hypointensity, and the distinction between the annulus fibrosa and nucleus pulposus is often indistinct (Fig. 2).

**Table 3:** Summary of major findings of presented status or diseases on conventional MR imaging and MR bone imaging

	Modic type 2 change	Modic type 3 change	Disc herniation	Ossification of the posterior longitudinal ligament	Spondylolysis	Vertebral metastasis	Compression fracture	Spondylodiscitis	Hemangiomas
Conventional MR findings	Hyperintensity on both T1/T2-weighted images	Hypointensity on both T1/T2-weighted images	Hypointensity on T2-weighted images Protusion/extrusion of the disc	Plate-like hypointensity on both T1/T2-weighted images	Often difficult to identify	Focal or diffuse signal changes May show pathological compression fracture	Changes in both signal and contour New lesions show cortical destruction and hyperintensity on fat-suppressed T2-weighted images	Disc space narrowing and endplate obscuration Bone marrow: hypointensity on T1-weighted images and hyperintensity on T2-weighted images Abnormal contrast enhancement	Hyperintensity on T2-weighted images Lipid-like hyperintensity on on T1-weighted images
MR bone imaging findings	Decrease in trabeculae	Sclerotic change	Visualization of the OPLL (disrupted or spared)	Plate-like bony structure	Defect in the pars interarticularis like CT	Even small destructive or sclerotic lesion visualized	Impaction of trabeculae in new lesions Osteosclerotic changes in old lesions	Early depiction of endplate changes Demonstration of abnormalities of all compartments on a single image	Thickened trabeculae (like “polka-dot” sign on CT) with a clear margin

*OPLL* ossification of the posterior longitudinal ligament

### Degenerative diseases

#### Modic type 2 change

**Fig. 3:**
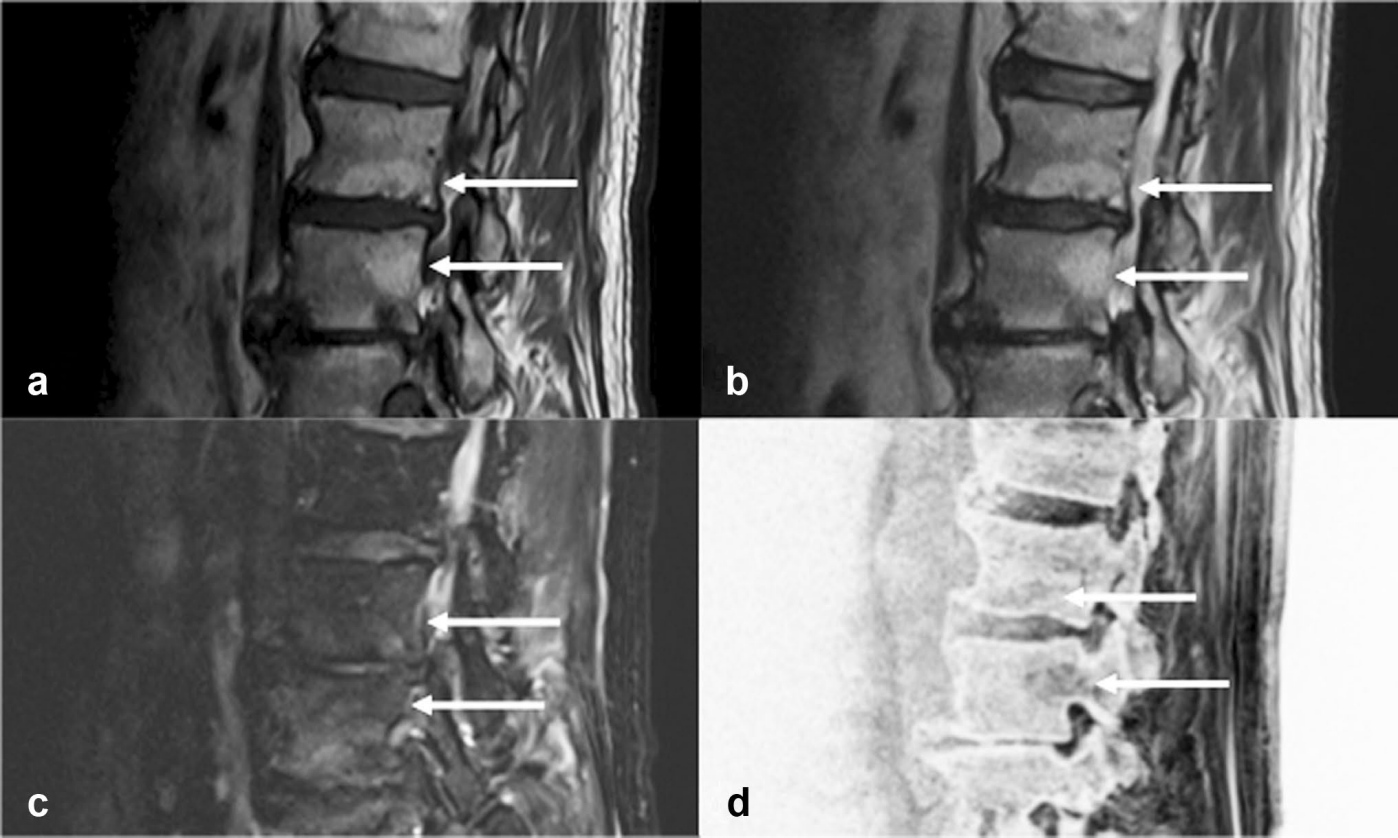
Modic type 2 change. Modic type endplate changes represent a classification for vertebral body endplate signal changes on MR imaging. Modic type 2 change represents red hemopoietic bone marrow conversion to yellow fatty marrow due to bone marrow ischemia. Sagittal T1-weighted (**a**), T2-weighted (**b**), and fat-suppressed T2-weighted (**c**) MR images of an 84-year-old male patient show signal changes compatible with fatty changes in the bone marrow (arrows). Sagittal MR bone image **d** shows sparse trabeculae (arrows)

Modic-type changes are alterations in the signal of the vertebral body endplate and adjacent area on MR imaging [27]. Modic type 2 change represents red hemopoietic marrow conversion into yellow fatty marrow due to bone marrow ischemia. MR bone images show type 2 changes as areas of decreased trabeculae due to degeneration (Fig. 3).

#### Modic type 3 change

**Fig. 4:**
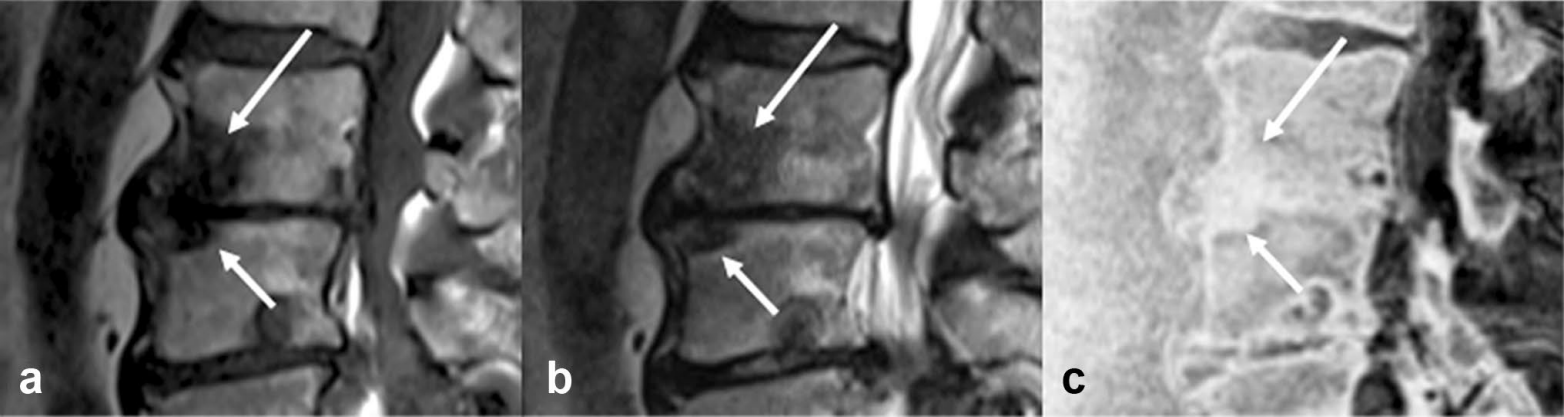
Modic type 3 change. Modic type 2 change represents subchondral bony sclerosis. Sagittal T1-weighted (**a**) and T2-weighted (**b**) MR images of an 84-year-old male patient show areas of hypointensity (arrows). Sagittal MR bone image **c** shows sclerotic changes as hyperintense areas (arrows)

Modic type 3 change represents subchondral bony sclerosis. On conventional MR sequences, this change is visualized as a hypointensity due to decreased protons, whereas it is visualized as an area of sclerosis on MR bone imaging (Fig. 4). Reliable detection of sclerosis in Modic changes has been reported using SWI-based MR bone imaging [28].

#### Disc diseases

**Fig. 5:**
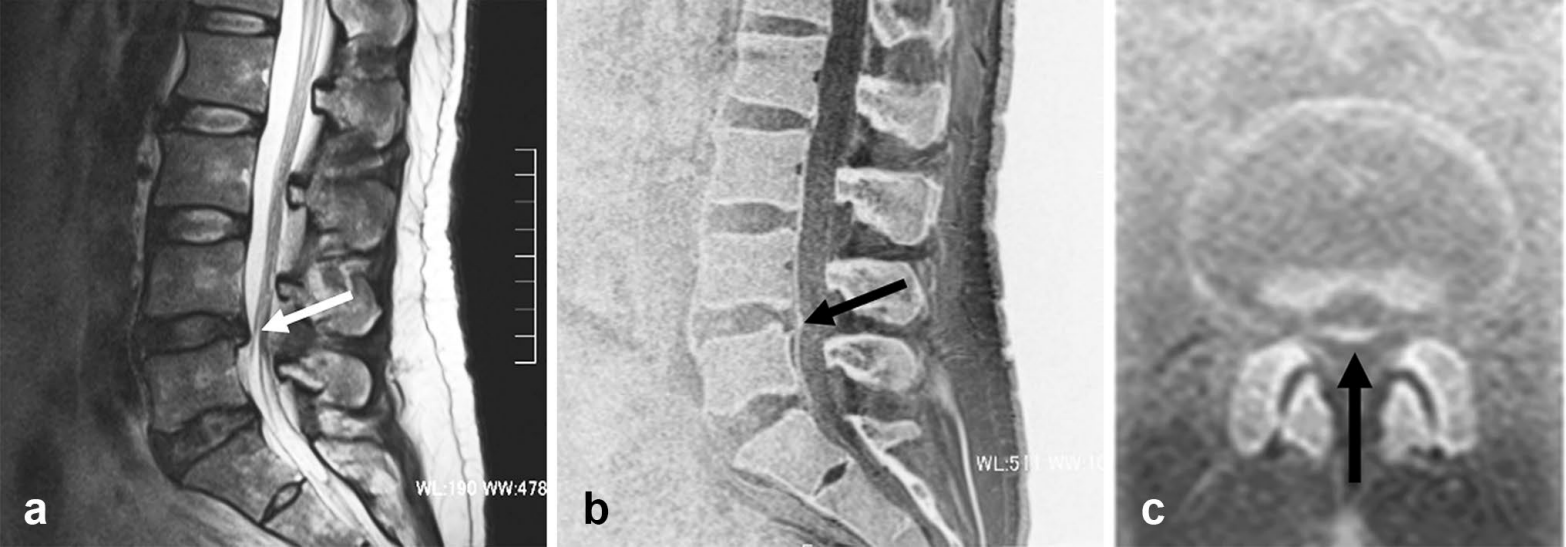
Lumbar disc herniation. Sagittal T2-weighted MR image **a** shows a herniated L4-5 disc in a 45-year-old male patient (arrow). Sagittal and axial MR bone images (**b** and **c**) show the herniated disc and preserved posterior longitudinal ligament (arrow)

For many years, conventional MR imaging has played a significant role in the diagnosis of vertebral disc diseases. The value of SWI-based MR bone imaging for the differentiation of osteophytes and disc herniations in patients with spinal radiculopathy has been reported as stated above [19]. Furthermore, ZTE MR bone imaging reportedly allows clear visualization of cervical neural foraminal stenosis caused by osteophytes, as well as disc herniation [21]. A perforation of the posterior longitudinal ligament along with that of the dura matter could be associated with surgical difficulty and, in some cases, intradural disc herniation resulting in unfavorable postoperative functional prognosis [22]. As MR bone imaging is likely to effectively demonstrate the status of the posterior longitudinal ligament in patients with disc herniation, it could serve as a valuable imaging technique preoperatively (Fig. 5). However, the diagnostic value of MR bone imaging in the preoperative assessment of the posterior longitudinal ligament still needs further verification in comparison with intraoperative findings.

#### Ossification of the posterior longitudinal ligament

**Fig. 6:**
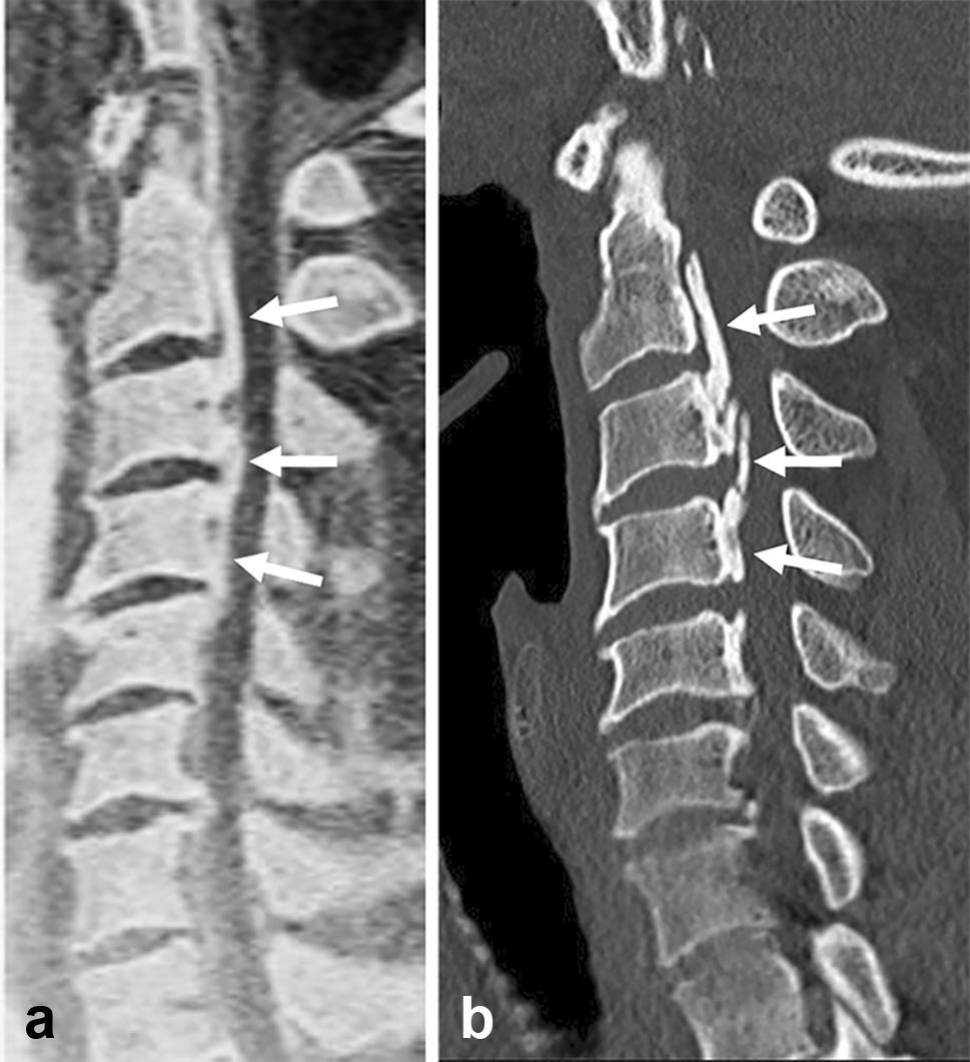
Ossification of the posterior longitudinal ligament. In diagnosing ossification of the posterior longitudinal ligament (OPLL), it is imperative to precisely assess the degree of ossification that causes spinal cord compression. Sagittal MR bone image **a** shows segmented OPLL at C2-C4 extending partially to C5 (arrows) in a 43-year-old male patient. Sagittal CT image **b** confirms the OPLL (arrows)

As it is caused by ossification of the ligament, ossification of the posterior longitudinal ligament (OPLL) can be demonstrated well by MR bone imaging with the agreement of CT, which can work effectively for preoperative evaluation, by obviating radiation exposure. Previous reports have described the value of MR bone imaging using ZTE and synthetic CT images for the diagnosis of OPLL [23, 29]. Like ZTE and synthetic CT images, the 3D-GRE sequence provides sufficient diagnostic imaging in the evaluation of ossification in the posterior longitudinal ligament (Fig. 6).

#### Spondylolysis

**Fig. 7:**
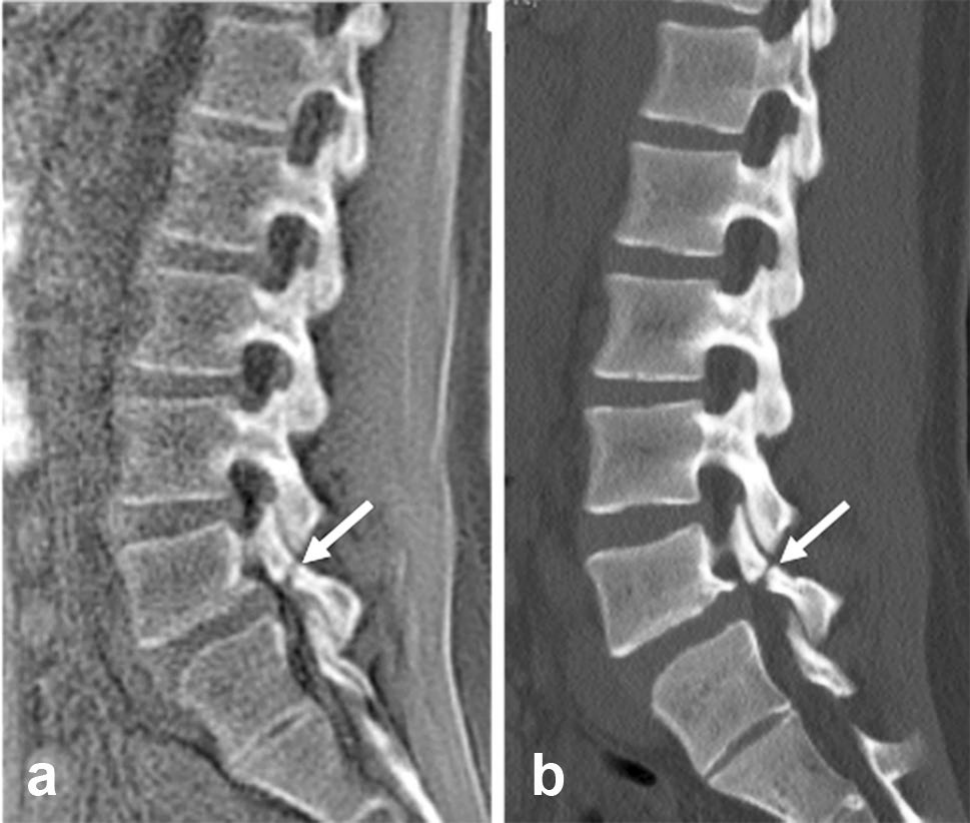
Spondylolysis. Spondylolysis is a defect in the pars interarticularis of the neural arch often seen in the adolescent population. Sagittal MR bone image **a** obtained with the ZTE sequence at 1.5 T shows a defect in the pars interarticularis of L5 in a 19-year-old female patient (arrow). Sagittal CT bone image **b** shows the same finding (arrow). (Courtesy of Akira Fujikawa, MD, PhD, Self-Defense Forces Central Hospital, Tokyo, Japan)

Spondylolysis is a defect in the pars interarticularis of the neural arch. Although it remains asymptomatic in most cases, it is one of the common causes of low back pain in adolescents. CT has long been the gold standard to demonstrate the pars interarticularis defect called the “Scottie dog sign” on oblique plain radiography, which can be well shown clearly by MR bone imaging without radiation exposure (Fig. 7).

### Tumors and tumor-like lesions

#### Vertebral metastasis

**Fig. 8:**
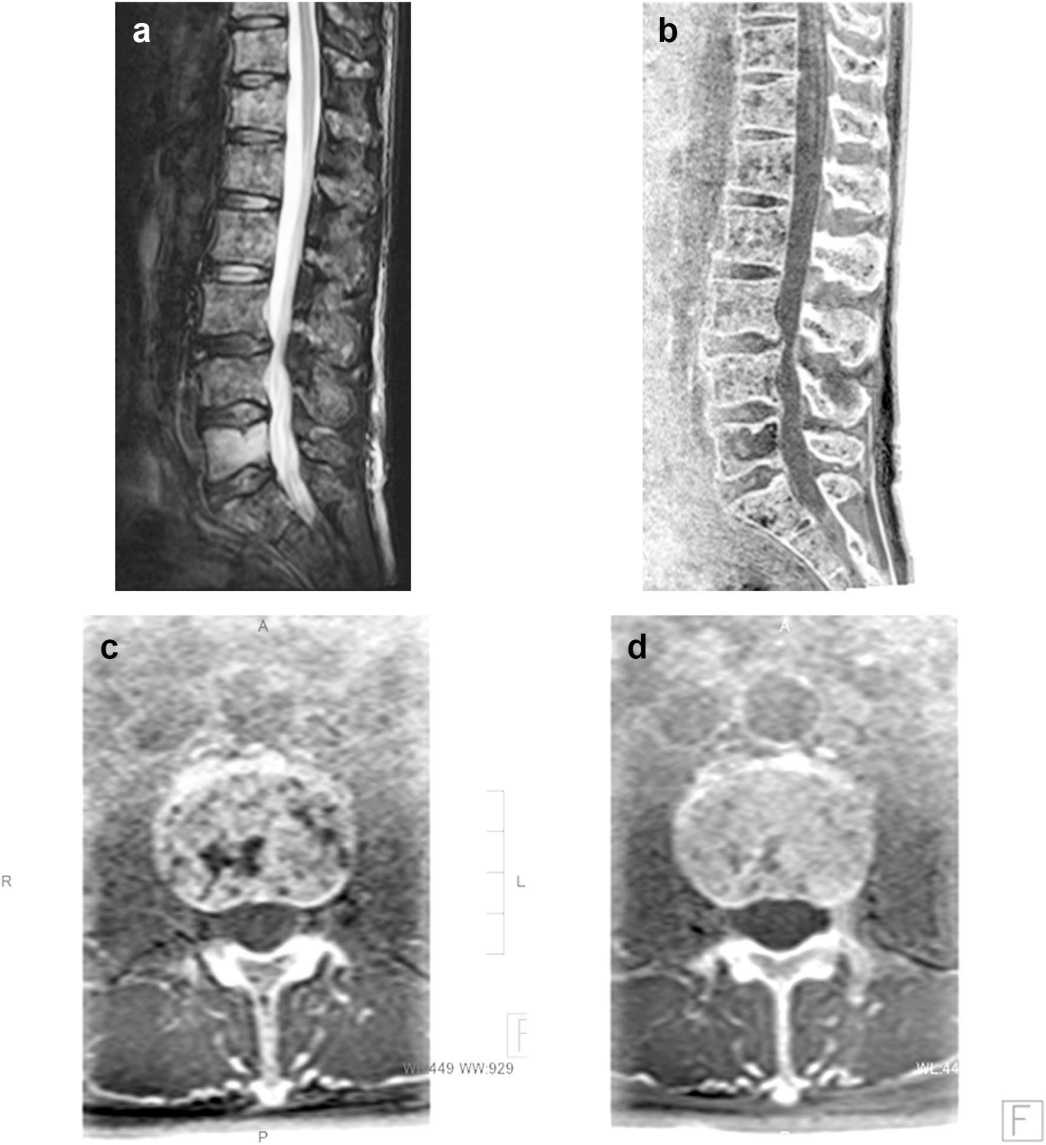
Plasma cell myeloma. Spinal metastasis is one of the common complications of plasma cell myeloma and often develops multiple destructive lesions. Sagittal fat-suppressed T2-weighted MR image shows numerous hyperintense lesions in the lumbar vertebrae and sacrum in a 60-year-old male patient (**a**). A mild compression fracture of the L5 body is also noted. Sagittal and axial MR bone images (**b**) and (**c**) show the lesions more clearly. Axial MR bone image (**d**) obtained at the same level after 3.5 months of chemotherapy shows the resolution of the lesions

Attempts to detect vertebral metastases by MR imaging are made during screening or searching for the cause of some neurological symptoms in patients with cancer. MR bone imaging may add little to conventional MR imaging, which itself has sufficient diagnostic capabilities, but can often demonstrate details of metastases and depict tiny lesions. Multiple well-circumscribed and lytic bone lesions of plasma cell myeloma (multiple myeloma) are clearly shown at initial diagnosis by MR bone imaging, which is also of diagnostic value in the follow-up of such patients (Fig. 8). As MR bone imaging generates contrast like that of CT, it may have more diagnostic value to detect osteosclerotic metastases, which are not easily detected on conventional MR imaging. The capability of MR bone imaging for metastatic vertebral lesions, compared with conventional MR imaging including diffusion-weighted imaging, has not yet been thoroughly established and awaits future assessment.

## Fractures

### Compression fracture

**Fig. 9:**
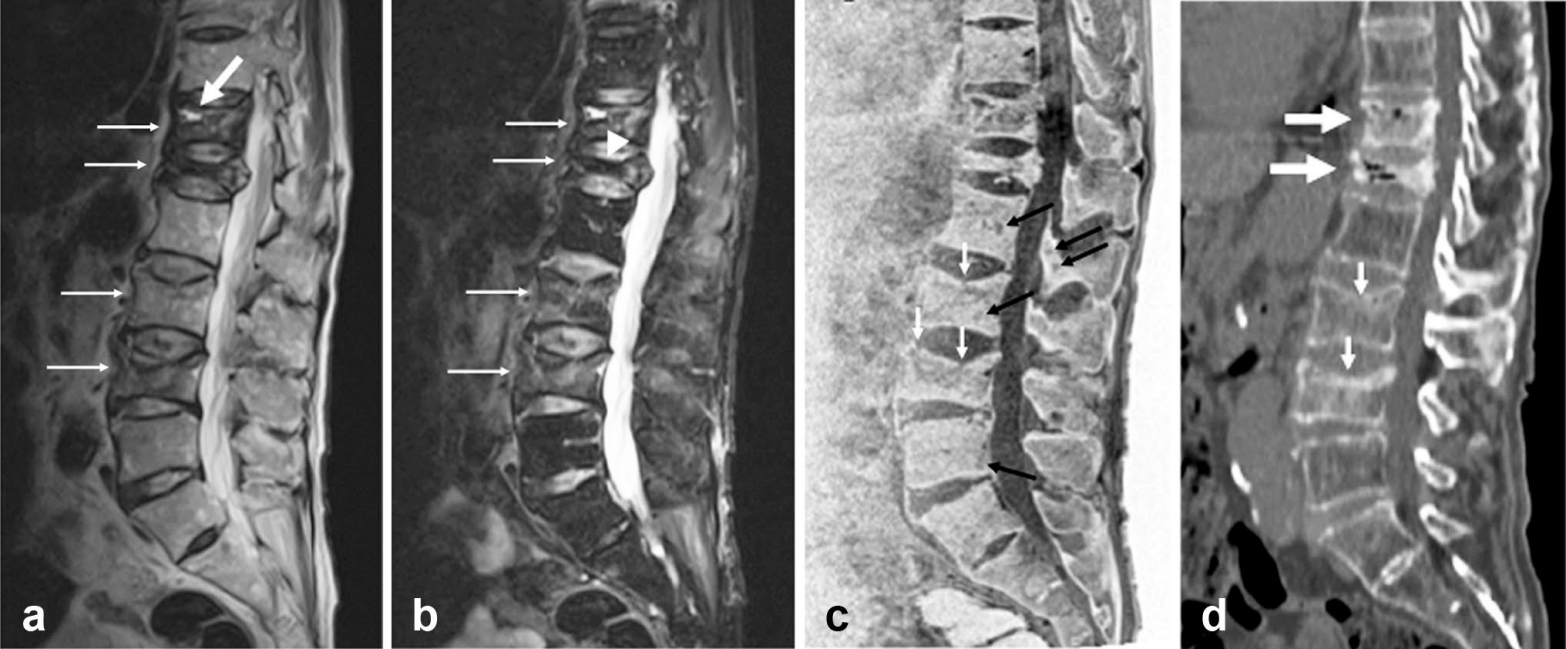
Recent pathologic compression fractures associated with old compression fractures. Sagittal T2-weighted MR image **a** shows compression fracture of Th11, Th12, L2, and L3 in a 77-year-old male patient with lung cancer (small arrows). Fluid sign is noted in the Th11 fracture (large arrow). Sagittal fat-suppressed T2-weighted MR image **b** shows the multiple fractures (arrows) and hypointensity in most parts of Th12, indicating old fracture (arrowhead). The hyperintensity of L2 and L3 suggests acute fracture. Part of Th11 also shows hyperintensity, suggesting recurrent acute fracture. On sagittal MR bone image **c**, the collapsed Th12 shows hyperintensity due to sclerosis. A tiny disruption of the cortex of L2 and L3 suggests a recent fracture (white arrows). Small hypointense lesions are probable metastatic foci from lung cancer (black arrows). Sagittal CT image **d** shows a sclerotic change of Th11 and Th12 as well as air indicating old fractures (large arrows). It also shows fracture of L2 and L3 with disruption of the cortex that is compatible with recent fracture as suggested on MR bone imaging (small arrows)

Although CT readily visualizes vertebral compression fractures, conventional MR imaging has also played a significant role, as it enables distinguishing acute compression fractures from other stages of fracture and demonstrates secondary changes in the spinal cord and nerve roots. MR bone imaging depicts the cortex and trabeculae of the vertebral body. Compression fractures with cortical destruction can be judged relatively new (Fig. 9). MR bone imaging allows visualization of the impaction of trabeculae, which also indicates new fractures. Osteosclerotic changes in old compression fractures are also well visualized by MR bone imaging.

Differentiation between osteoporotic and pathological fractures in the acute phase is often clinically challenging. The following features favor the diagnosis of a benign compression fracture: no bony destruction, preserved fatty bone marrow signal on T1-weighted imaging, hypointensity band on T1- and T2-weighted imaging indicating a fracture line, the “fluid sign” indicative of vertebral collapse, retropulsion of the posterosuperior cortex of the vertebral body, no epidural mass, and multiple compression fractures [30]. MR bone imaging shows even small metastatic foci in the vertebral bodies and in the posterior elements that directly suggest the presence of metastasis, which aids in establishing the diagnosis of pathological compression fractures caused by vertebral metastases along with other findings on conventional MR imaging (Fig. 9).

Regarding other traumatic vertebral injuries, SWI-based MR bone imaging reportedly shows better diagnostic performance than conventional MR imaging in identifying posterior cortex involvement, fracture lines, and cortical breaks (sensitivity 0.86–0.98, specificity 0.99–1.00) [16]. Another article reports a better diagnostic value of T1-weighted 3D GRE sequence than UTE for fracture detection (sensitivity, specificity, and accuracy were 0.95, 0.98, and 0.97 for T1-weighted 3D GRE and 0.91, 0.96, and 0.95 for UTE) [26].

### Infectious diseases

**Fig. 10:**
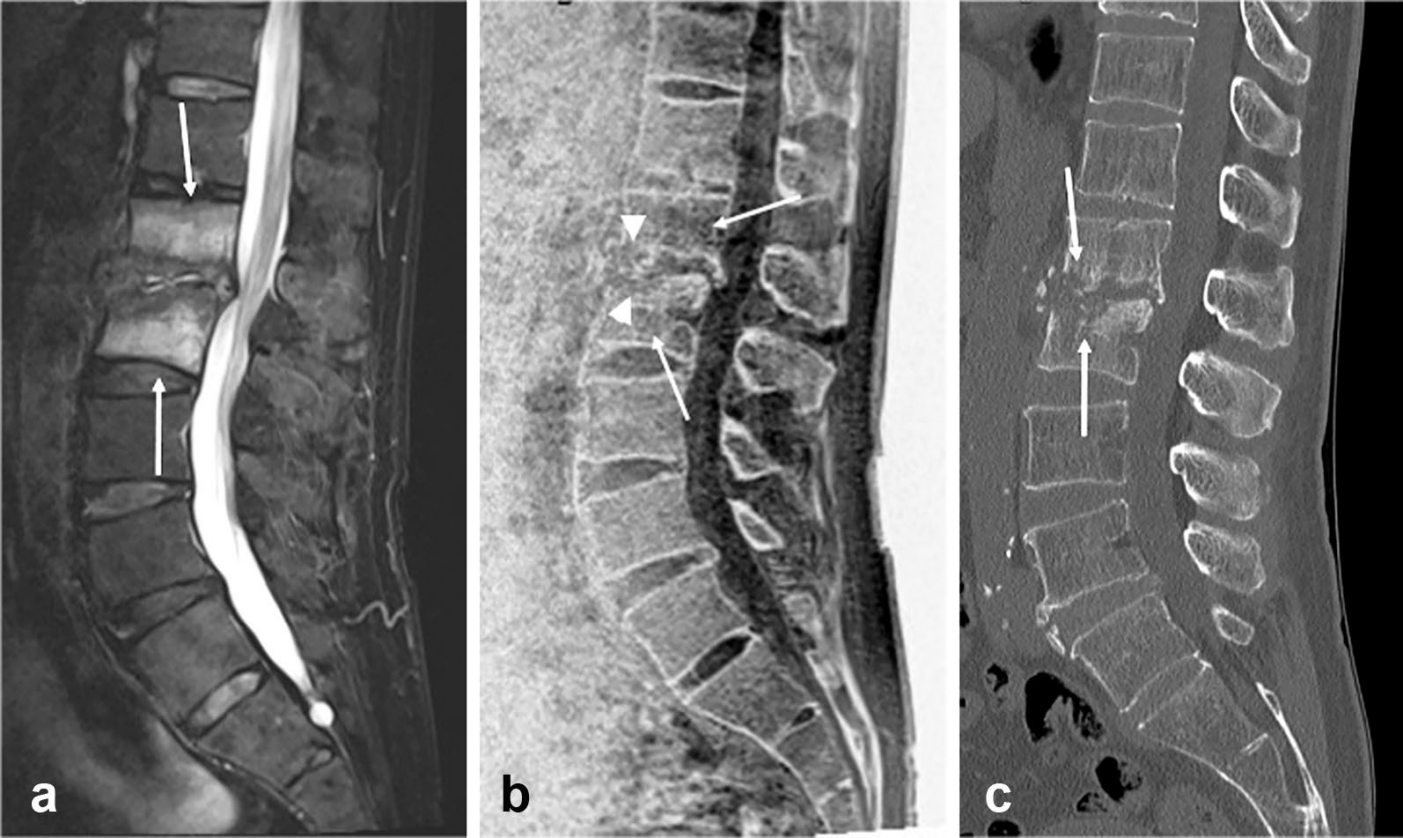
Pyogenic spondylodiscitis. Sagittal fat-suppressed T2-weighted MR image (**a**) of a 64-year-old male patient shows abnormal hyperintensity in L1 and L2 vertebrae and the flattened L1-2 disc due to spondylodiscitis (arrows). Sagittal MR bone image (**b**) clearly shows the disrupted endplates of L1 and L2 vertebrae (arrowheads) and sparse trabeculae (arrows). Sagittal CT image (**c**) shows the corresponding destruction of the endplates of the L1 and L2 bodies facing the narrowed L1-2 disc (arrows)

Spondylodiscitis refers to an infection involving the intervertebral discs and adjacent vertebrae. As known, it affects the vertebral endplate in the early phase, followed by disc involvement, and in some cases, abscess formation in the spinal epidural space or psoas muscle. As the disease progresses, the disc space narrows and the endplate becomes ill-defined. These pathological processes are better depicted on CT. MR imaging more sensitively visualizes not only the disc space and endplate abnormalities but also signal changes that develop in the vertebrae and intervertebral discs. Contrast enhancement in these structures on postcontrast MR imaging reflects disease activity. MR bone imaging is a modality that can visualize these changes in a single image. As previously reported using ZTE MR bone imaging in pediatric patients with miscellaneous lesions [31], changes due to infection or inflammation may be equally or better visualized on MR bone imaging when compared with CT or conventional MR imaging (Fig. 10). Therefore, in addition to conventional MR sequences, including postcontrast imaging, MR bone imaging may play a significant role in the diagnosis of spondylodiscitis in its early phase.

### Hemangioma

**Fig. 11:**
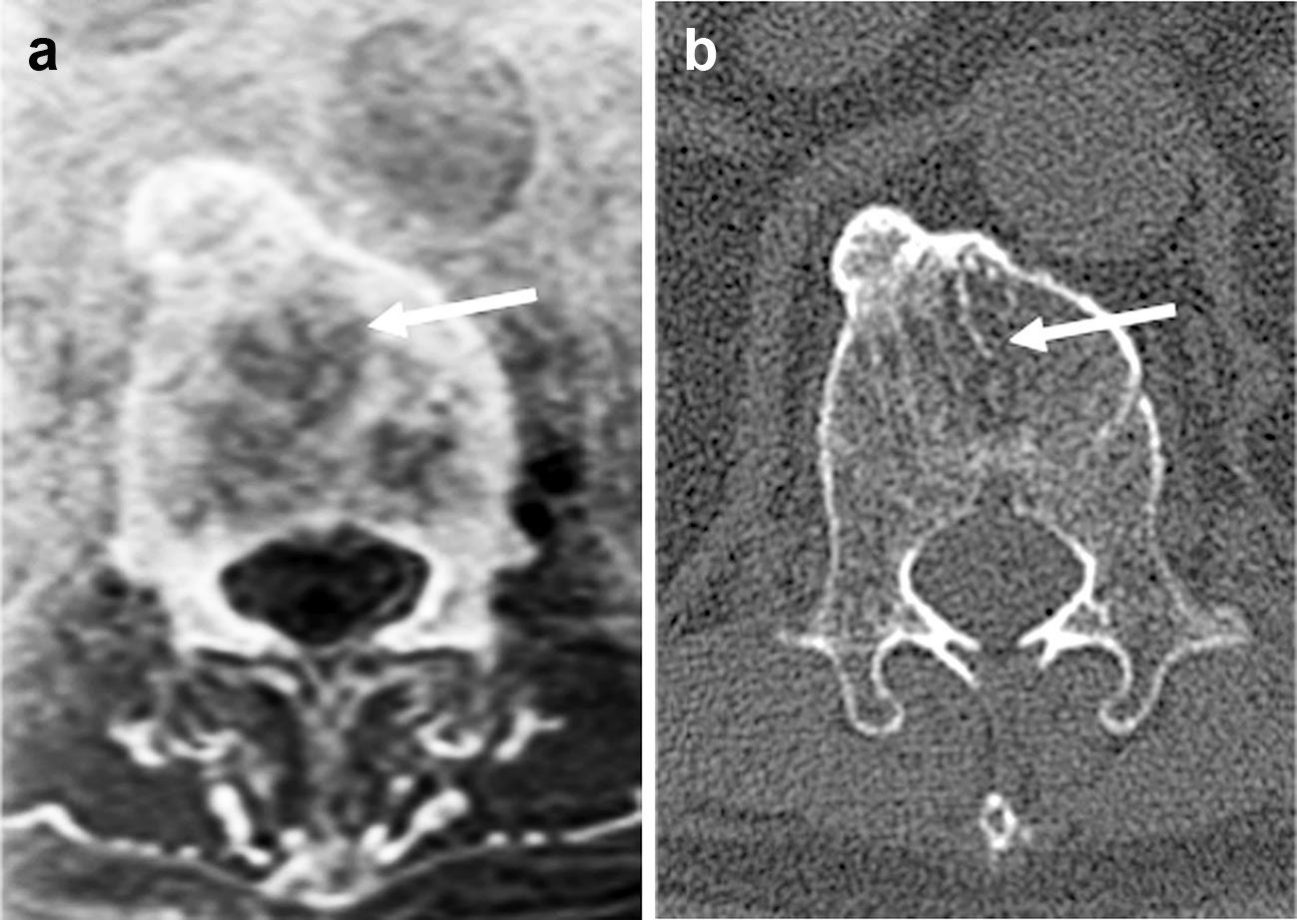
Hemangioma. Axial MR bone image (**a**) of an 84-year-old male shows a lucent lesion margin (arrow). Thickened internal trabeculae are also well visualized. Axial CT image (**b**) shows similar findings, but the lesion margin is unclear (arrow)

Vertebral hemangiomas are often asymptomatic and incidentally found on CT or MR imaging. They are detected in about 10% of adults and are often multiple. Capillaries or spongy vascular spaces lined with thin endothelium are present in the interstices of tissues consisting of sparse and thickened trabecular bone and adipose tissue that has replaced hematopoietic tissue. It is not a true neoplasm, but a “benign” lesion that should be called a vascular malformation. They have large vascular lumina and less fatty stroma. They could form mass lesions inside and outside the vertebral body and cause pathological fractures. On CT, thickened vertebral trabeculae show a “polka-dot” sign. On MR imaging, they are seen as areas with increased fat localized in the bone marrow and as hyperintensities on T2-weighted images. MR bone images demonstrate the lesion margin and thickened internal trabeculae, resulting in a good depiction of the imaging characteristics of vertebral hemangiomas (Fig. 11). Other vertebral anomalies, especially in children, are expected to be effectively visualized by MR bone imaging without radiation exposure.

## Discussion

CT has been considered the best modality for the visualization of cortical bone. The value of MR bone imaging to visualize bone has recently been shown in many areas of the body. Some previous reports have shown statistically good to excellent agreement between the two modalities [11, 12, 32, 33].

In this article, we have presented several MR bone imaging techniques. High-resolution 3D GRE sequences are used widely along with UTE/ZTE sequences. Many previous reports show that ZTE/UTE sequences as well as black bone imaging depict the bone cortex well. Accordingly, we can obtain volume-rendered 3D images of the spine and other bony structures as in CT. On the other hand, 3D GRE sequences are excellent at delineating the internal structures of the vertebral bodies, as illustrated by the cases presented in this article. It seems appropriate that both be used properly and effectively according to the suspected pathology. It is advisable to choose black bone imaging or UTE/ZTE imaging to demonstrate cortical bone abnormalities, while a 3D GRE sequence can be a better choice to obtain both bone structural information and information on adjacent soft tissue such as ligaments and muscles [34].

Through presenting a variety of cases, we show that thanks to its unique inherent imaging capability, as stated above, MR bone imaging appears to have the ability to allow vertebral diseases and associated soft tissue lesions to be visualized clearly and is comparable or superior to conventional CT in terms of the spatial resolution and contrast it offers. For the diagnosis of vertebral metastases, vertebral hemangioma, and some degenerative lesions such as OPLL, MR bone imaging could replace CT. However, for other pathologies, MR bone imaging can still not replace CT or conventional MR imaging sequences but is a useful adjunct to such imaging techniques. It is worth mentioning that conventional MR imaging techniques like T1-weighted, T2-weighted, fat-suppressed T2-weighted imaging, MR angiography, and water-sensitive imaging [10, 26], which yield information on the spinal cord and associated adjunct lesions, can be obtained along with the bone images when we employ MR bone imaging. Moreover, as many reports have already pointed out, one of the values of MR bone imaging is that it allows radiation exposure to be avoided, especially in children [35–37].

Recent advances in image postprocessing techniques have greatly improved the fusion of images generated from multiple imaging modalities. Fusion of CT and MR images is providing images of value not only for the diagnosis but also for treatment guidance and surgical navigation [38–40]. With MR bone imaging, visualization of soft tissues and bones can be obtained in one session with similar body geometry, offering new perspectives for diagnosis, treatment planning, and guidance [7].

It may be worth mentioning some disadvantages of MR bone imaging. Compared with CT, the scanning time for MR imaging including MR bone imaging is long. The addition of MR bone imaging to conventional sequences would further extend the total scanning time. It may be a drawback, especially in children. Another disadvantage is susceptibility artifacts that are moderate to severe depending on the scanning technique (Table 1). Moreover, due to the inherent features to produce signals of bones, it often occurs that air like that in the paranasal sinus or blood-breakdown products within lesions can show signal intensity indistinguishable from bones. When such findings are suspected, referring to conventional MR images may be required.

In conclusion, MR bone imaging, which has become an adjunct to conventional MR imaging as a new technique, provides a unique contrast of bones from the surrounding tissues and can be used effectively in conjunction with CT.
